# Interferometric rotating point spread function

**DOI:** 10.1038/s41598-017-06203-1

**Published:** 2017-07-19

**Authors:** Wei Wang, Guohai Situ

**Affiliations:** 1 Shanghai Institute of Optics and Fine Mechanics, Chinese Academy of Sciences, Shanghai, 201800 China; 2 University of Chinese Academy of Sciences, Beijing, 100049 China

## Abstract

Rotating point spread functions (PSF), such as the double helix (DH) PSF, are widely used in localization-based super-resolution imaging because of their large working depth range. In this article, we propose an interferometric DH PSF (iDH PSF) using two opposed objective lenses as in the 4Pi microscope. In the proposed iDH PSF, the super-resolution in the axial PSF is transferred to the azimuthal rotation. Moreover, we design an iDH PSF whose imaging range reaches 3 *μm*, which is roughly 3 times as much as that which can be obtained by using other interferometric localization-based super-resolution imaging methods.

## Introduction

Localization-based super-resolution imaging methods^1–5^, such as the photoactivated localization microscopy (PALM) and the stochastic optical reconstruction microscopy (STORM), have been demonstrated to have the capability of overcoming the diffraction limit in three dimensions. In these methods, the resolution is determined by the localization precision rather than the diffraction limit. For PALM and STORM, it has been shown that engineering the PSF of the imaging system by using the astigmatic point spread function (PSF)^2^, double helix PSF (DH PSF)^3,6,7^, and Airy PSF^8^ can enable a high localization precision over a large depth range. Up to now, the DH PSF has been widely used in localization-based super-resolution imaging methods and localization-based single particle tracking methods because of its large imaging depth and simplicity in extracting the emitter’s 3D position. However, anisotropy in the localization process affects the axial precision, making it worse than the transverse precision^6^.

**Figure 1:**
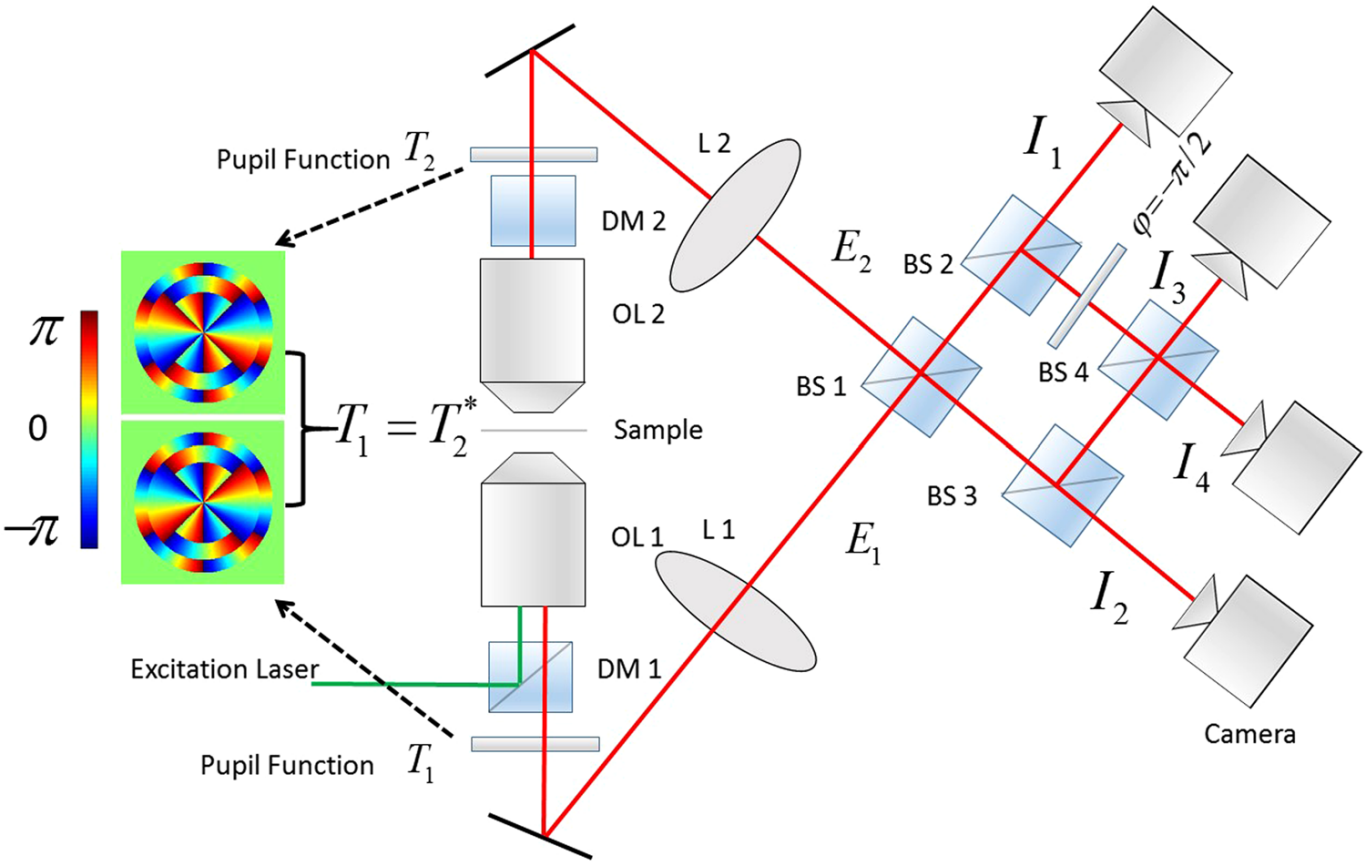
Schematic illustration of the setup for generating interferometric rotating PSF. In the interferometric detection scheme, the light fields *E*_1_, *E*_2_ collected by objective lenses OL1 and OL2 are modulated by conjugate pupil functions *T*_1_ and *T*_2_ (${T}_{1}={T}_{2}^{\ast }$) respectively. The beam splitters (BS) and an constant phase retardation plate before BS 4 combines the light fields to produce four phase-shifted interferometric signals *I*_1_, *I*_2_, *I*_3_, *I*_4_ on the cameras. DM: Dichroic mirror, BS: beam splitter. Inset: pupil functions (DH-P3) for generating the DH PSF. In the non-interferometric detection scheme, only one objective lens OL 1 is used, and the four beam splitters are removed from the detection path.

In this manuscript, we propose a new kind of rotating PSF: the interferometric DH PSF (iDH PSF). As shown schematically in Fig. 1, the two lobes in the coherent DH PSFs collected by the two objective lenses overlap perfectly in the proposed iDH PSF, thus allowing the interference in the azimuthal direction. We will show that the axial information is encoded in the azimuthal direction in iDH PSF. The interference between the waves collected by the two opposed objective lenses in 4Pi imaging improves the axial resolution by a factor of 3–7 compared with using a single objective lenses^9–12^. Therefore, iDH PSF based on interferometric detection schemes^13–18^ shows super-resolution in the azimuthal direction, and side lobes occur in the original lobe position which is similar with the axial super-resolution and axial side bands in 4Pi imaging. Further, we show that localization-based super-resolution imaging using iDH PSF improves the transverse precisions by two-fold, and the axial localization precision by a factor of 4-5 in comparison with using DH PSF. Thus, the anisotropy in localization precisions using DH PSF is greatly improved by using iDH PSF. Moreover, we design an iDH PSF to enable 3D imaging over a 3 *μm* depth range which is around 3 times of other interferometric localization based super-resolution methods^13–15^.

## Results and Discussions

Figure 1 shows the setup of the fluorescence imaging system that employs the proposed technique. Throughout the article, the numerical aperture (NA) of both the objective lenses is 0.95, and the wavelength *λ* is 532 nm. In the scalar approximation (neglecting the vector character and polarization of the light field), the coherent PSF using a single objective with maximum light collection angle Θ has the following form ref. 19

1 $$\begin{array}{rcl}E({\rho }_{p},z,{\varphi }_{p}) & = & -\frac{ik}{2\pi }{\int }_{0}^{2\pi }d\varphi {\int }_{0}^{{\rm{\Theta }}}d\theta T(\theta ,\varphi )\sin \,\theta \sqrt{\cos \,\theta }\exp \,(ikz\,\cos \,\theta )\\  &  & \times \,\exp (ik{\rho }_{p}\,\sin \,\theta \,\cos \,({\varphi }_{p}-\varphi )),\end{array}$$

where *T* represents the pupil function, *k* is the wavenumber, and (*θ*, *ϕ*), (*θ*, *ϕ*_*p*_) are the polar coordinates in the object plane and the image plane, respectively. For fluorescence imaging, the detection measures the intensities, |*E*|^2^.

For the pupil function *T*(*θ*, *ϕ*), we adopt the one proposed by Prasad^20^, and divide the pupil into *L* annular zones, with successive zones carrying spiral phase profiles of successively larger topological charge *l*:

2 $$T(\theta ,\varphi )=\{\exp (il\varphi )|\theta \in [{\theta }_{l},{\theta }_{l+1}],\quad l=1,\ldots ,L\},$$

where *θ*_*l*_, *θ*_*l*+1_ are the lower and the upper bounds of the *l*^*th*^ annular zone, respectively^20^. The size of every annular zone is flexible in principle. In our study, we divide the aperture *T*(*θ*, *ϕ*) equally with respect to *θ* for simplicity. For this pupil function, the coherent PSF in Eq. (1) has the following analytic form:

3 $$E({\rho }_{p},z,{\varphi }_{p})=-\sum _{l\mathrm{=1}}^{L}{i}^{l+1}k{\int }_{{\theta }_{l}}^{{\theta }_{l+1}}d\theta \,\sin \,\theta \sqrt{\cos \,\theta }\exp [i(kz\,\cos \,\theta -l{\varphi }_{p})]\,{J}_{l}(k{\rho }_{p}\,\sin \,\theta ).$$

**Figure 2:**
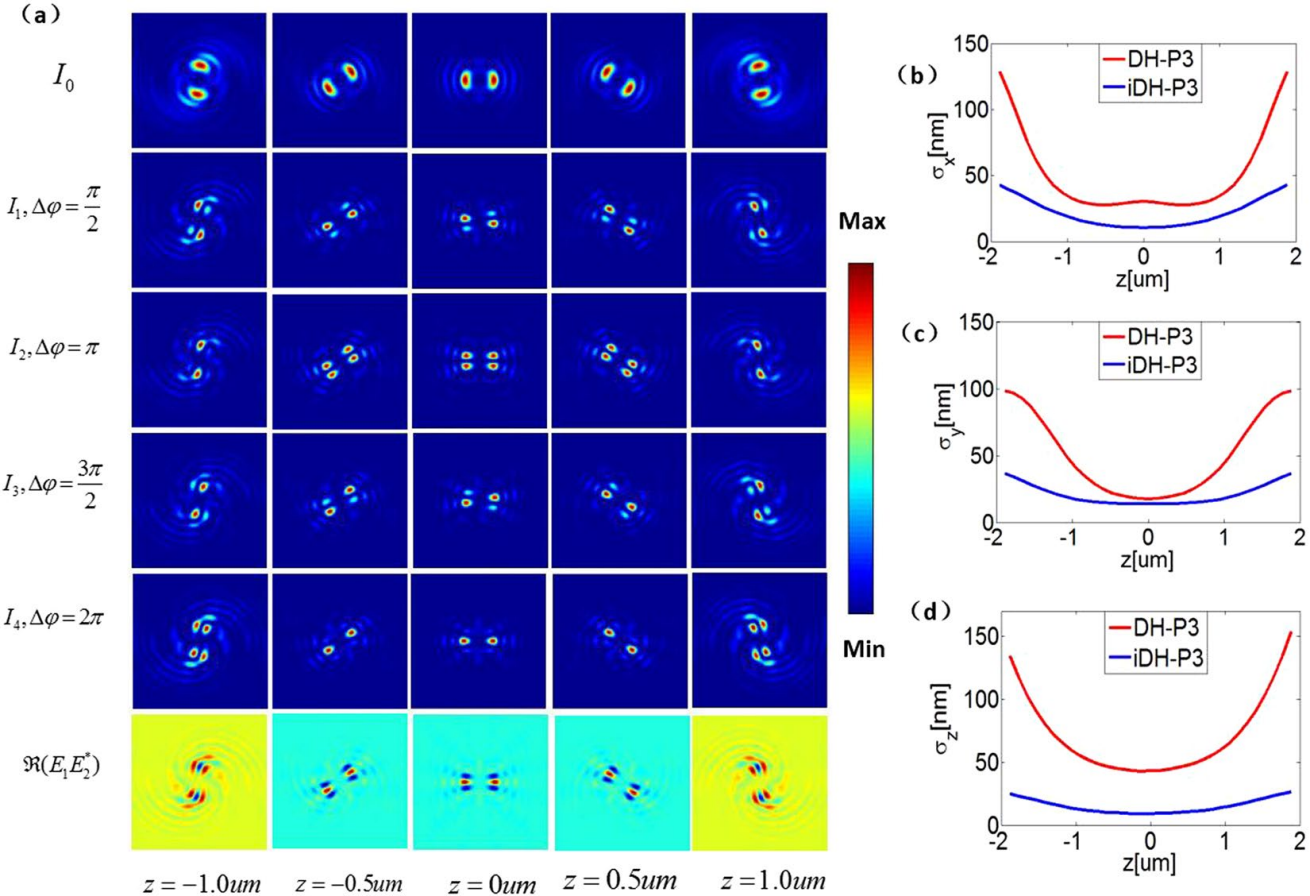
(**a**) Top row: the DH PSF using the non-interferometric detection scheme; Middle four rows: Four phase-shifted intensity images *I*_1_, *I*_2_, *I*_3_, *I*_4_ recorded by the four cameras using the interferometric detection scheme, which clearly exhibit the rotation of the pattern as the defocus changes. Bottom row: iDH PSF: iDH-P3 ($\Re ({E}_{1}{E}_{2}^{\ast })$). In comparison with the DH PSF, the size of the main lobe in the iDH PSF is significantly decreased because the interference effect occurs in the azimuthal direction. In addition, the iDH-PSF rotates across the same angle as the DH PSF at the same defocus. (**b**–**d**) Theoretical localization precision *σ*_*x*_, *σ*_*y*_, *σ*_*z*_ using the DH PSF and the iDH PSF, respectively.

The phase term exp [*i*(*kz* cos*θ* − *lϕ*_*p*_)] in Eq. (3) implies that the PSF rotates as defocus. Here we propose to generalize the annular pupil function in Eq. (2), and define the generalized topological charge *l* → *l*_1_ = *nl*, where *n* is an integer. A similar definition of the generalized topological charge was also reported in Yu’s work^21^. Different from the single lobe in the single helix (SH) PSF, we found that the number of main lobes in the PSF in this generalized form is equals to the ratio of *l*_1_ to *l*. Now let us consider the simplest case of *l*_1_ = 2*l*. The PSF (pixel size: 5.6 *u*m, image size: 100*100 pixels) in this case contains two main lobes as illustrated in Fig. 2(a). It is clearly seen that it looks similar to the DH-PSF^3,6,7^. As such, we refer to it as the DH-P *L* PSF due to the division of the pupil function. For instance, it can be denoted as DH-P3 PSF when the number of zones *L* = 3. For a typical microscope with one objective lens, the rotation over the defocus range (−1, 1) *μ*m is by the angle of *δϕ*_*p*_ = *π* for the DH-P3 PSF, as seen from Fig. 2(a).

Since the DH PSF encodes the axial information of the imaged points in the azimuthal direction^3,6,20,22^, it promises 3D imaging of sparsely distributed fluorescent particles without the need of scanning in the axial direction. Given a PSF and a noise level, the theoretical best-case precision in the *x*, *y*, *z* directions, (*σ*_*x*_, *σ*_*y*_, *σ*_*z*_), is determined by the Cramér-Rao lower bound (CRLB)^23^. For the DH-P3 PSF shown in Fig. 2(a), the average theoretical localization precision (*σ*_*x*_, *σ*_*y*_, *σ*_*z*_) = (29 nm, 25 nm, 48 nm) over the depth of 2 *μ*m in the case that the power of the signal is 1000 photons and the homogeneous background is 10 photons per pixel (with a pixel size 5.6 *u*m), as suggested by the theoretical results plotted in Fig. 2(b–d).

However, one disadvantage by using the DH-PSF is the anisotropy of localization precision: the axial precision *σ*_*z*_ is worse than the transverse ones^6^. This problem is also encountered in widefield imaging with large-NA objective lenses. This can be solved by the interferometric detection scheme using two objectives lenses, such as the 4Pi microscope^9,12^. We note that the interferometric detection scheme has been adopted in localization-based super resolution imaging. Methods such as the interferometric PALM (iPALM)^14^ and the 4Pi-SMS^13,15^ have been demonstrated to have better localization precision in comparison to those with a single objective lens. In our understanding, there are two main reasons causing this advantage: (i) The collected number of photons in the two-objective-lens system is doubled, resulting in the improvement of the localization precisions by a factor of $\sqrt{2}$. (ii) Super resolution occurs in the axial dimension due to the interference between the waves *E*_1_, *E*_2_ from the two objective lenses (see Fig. 1). Hence the anisotropy in the localization precision is reduced. In 2011, engineered PSF such as astigmatism has been introduced into iPALM^17^.

Here we introduce the interferometric detection scheme into the DH PSF. To make the two coherent DH PSF from the two objective lenses interfere, the two lobes in the DH PSF *E*_1_ and *E*_2_ must overlap. We note that the two DH PSFs will rotate in opposite direction if the pupil functions insert to modulate both the DH PSF have the same form, prohibiting the interference to occur. However, the two lobes in the two coherent DH PSFs will overlap if the corresponding pupil functions (*T*_1_, *T*_2_) are mutually conjugate, i.e.

4 $$\begin{array}{l}{T}_{1}(\theta ,\varphi )=\{\exp (i2l\varphi )|\theta \in [{\theta }_{l},{\theta }_{l+1}],\quad l=1,\ldots L\}\\ {T}_{2}(\theta ,\varphi )=\{\exp (-i2l\varphi )|\theta \in [{\theta }_{l},{\theta }_{l+1}],\quad l=1,\ldots L\},\end{array}$$

because the coherent PSFs satisfy the condition that ${E}_{1}=-{E}_{2}^{\ast }$ mathematically.

By inserting the above pupil functions into the setup of 4Pi-SMS^13^ shown in Fig. 1, we can generate the interferometric rotating PSF. In principle, the interferometric detection scheme records four phase-shifted intensity images *I*_*m*_, (*m* = 1, 2, 3, 4), by the four cameras, respectively:

5 $${I}_{m}={|{E}_{1}+\exp (i{\rm{\Delta }}{\phi }_{m}){E}_{2}|}^{2}$$

where Δ*φ*_*m*_ = (*m* − 1)*π*/2 is the phase shift. Experimentally, the reflected and the transmitted electric field emerging from the beam splitter (BS) have a phase difference *π*/2. Therefore, two *π* phase-shifted interference signal *I*_1_, *I*_2_ are collected. Through inserting an additional phase retardation (*φ* = −*π*/2) before BS 4, all four phase-shifted intensity images *I*_*m*_ can be detected. The figures in the four middle rows of Fig. 2(a) show the examples of the intensity images produced by using the two mutual-conjugated DH-P3 pupil functions described by Eq. (4). It is clearly seen that the two intensity images retain the same rotation property as the DH PSFs shown in the first row of Fig. 2(a), but the patterns of the PSF in this case exhibit fringe-like structures due to the interference effect. The positions of the patterns change as a function of the defocus and the phase shift. The iDH-PSF shown in the bottom row in Fig. 2(a) is defined as the intensity difference $\Re ({I}_{4}-{I}_{2}-i({I}_{3}-{I}_{1}))$ calculated from the phase-shifted images. To be specific, let us take the annular pupil function as an example. For this function, we have $\Re ({E}_{1}{E}_{2}^{\ast })=-\Re ({E}_{1}{E}_{1})$. Each of the the two main lobes in the DH PSF divides into three sub-lobes in the iDH-PSF, as plotted in the bottom row in Fig. 2(a). Because the size of the middle sub-lobe of the main lobes in the iDH PSF is only about 1/4 of that of the DH PSF, it is more easier to locate the position. That is where the better resolution in the azimuthal direction comes in, in a way similar to the 4Pi microscopy (increased by a factor of 3–7)^9–12^. The difference is that the axial dimension is encoded in the azimuthal direction in the proposed technique. That is to say, the iDH PSF technique transfers the effects exhibited in the axial direction in the 4Pi microscopy to the azimuthal direction. Although DH PSF has been demonstrated in interferometric detection schemes using mirror coverslips^16^, interferometric DH PSFs with three small lobes has not been reported to date.

The resolution improvement is demonstrated in Fig. 2(b–d), which plots the theoretical localization precision using the iDH-P3 PSF (iDH PSF with three annular pupil zones). In interferometric detection schemes, since we have multiple measurements *I*_*m*_ rather than one in non-interferometric setups, the model for calculating the CRLB from the four measurements *I*_*m*_ is different. Here we adopt the model proposed by Middendorf *et al*.^13^. Through using two objective lenses, the number of photons is increased from 1000 to 2000, while the background noise level does not change (10 photons per pixel). The results suggest that the iDH-PSF provides an average theoretical localization precision (13 nm, 15 nm, 11 nm) at the defocus of ±2 *μ*m for 1000 signal photons and 10 background photons. Comparing to the DH-PSF, the lateral localization precisions $\sqrt{{\sigma }_{x}{\sigma }_{y}}$ is increased by a factor of 2, and the axial one by a factor of 4.3. This indicates that the anisotropy in localization precision is reduced. Compared to the 4Pi-SMS, the improvement in the lateral dimension is slightly better, while the increase of the axial dimension is not that significant. It might be caused by the fact that the interferometric effect changes the shapes of the lobes in the radical and azimuthal direction simultaneously (mostly in the azimuthal direction).

**Figure 3:**
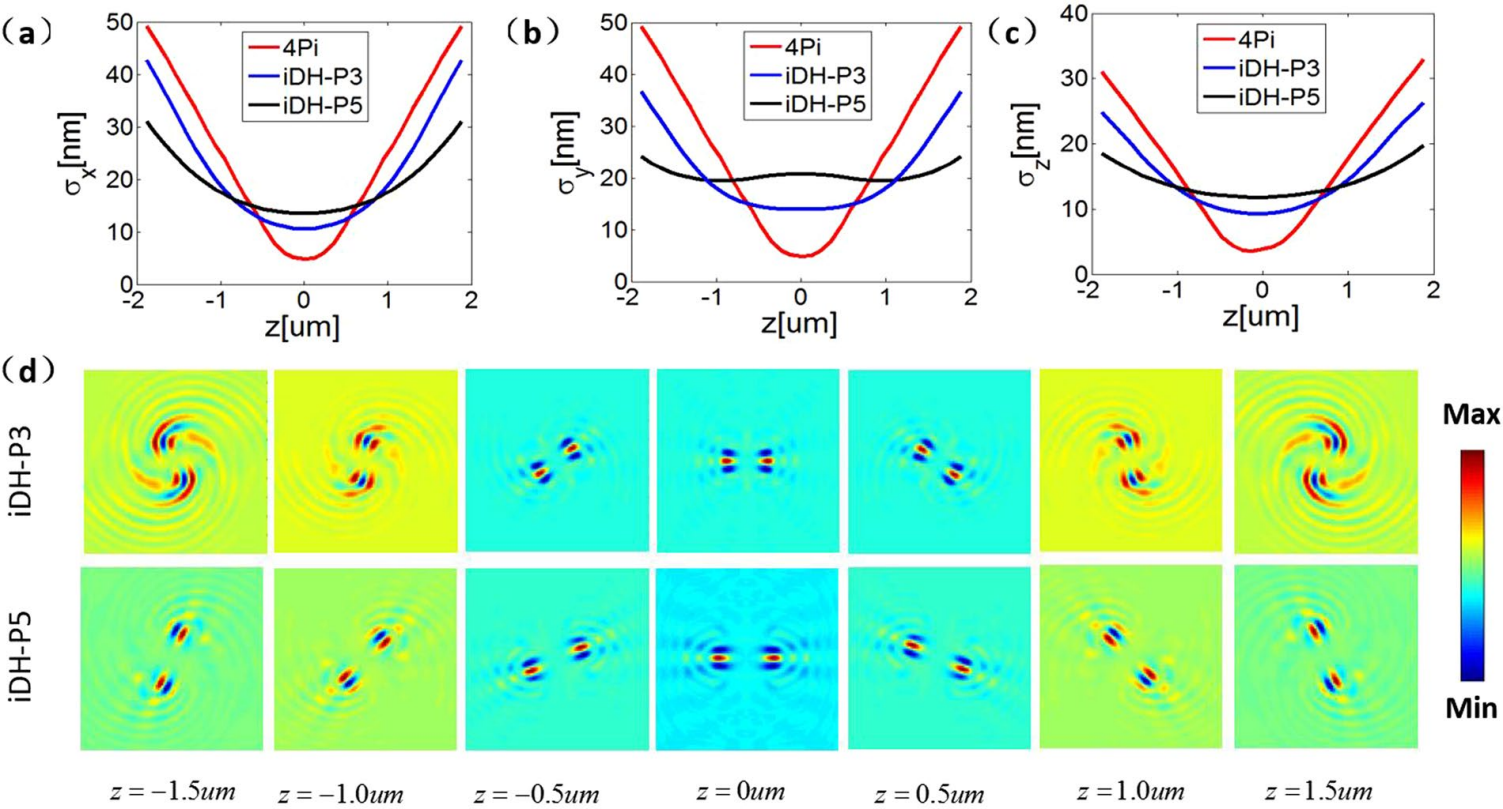
(**a**–**c**) Theoretical localization precision *σ*_*x*_, *σ*_*y*_, *σ*_*z*_ using the 4Pi, and the iDH PSF (iDH-P3, iDH-P5) respectively. The depth range is extended as indicated by the flatness of the curves. (**d**) Images of iDH PSFs (iDH-P3, iDH-P5) over the 3 *μ*m depth range.

One of the advantages the DH-PSF can offer in scan-less 3D imaging relies on its large depth range. The iDH-PSF inherits this property by nature. Traditional interferometric-localization-based super resolution imaging techniques such as the 4Pi-SMS provide a range of about 2*λ* (about 1 *μ*m)^15,18^. The localization precisions (*σ*_*x*_, *σ*_*y*_, *σ*_*z*_) using the 4Pi-SMS and the iDH-PSF of different forms are plotted in Fig. 3(a–c), respectively. The results suggest that a larger depth range can be achieved via the iDH-PSF scheme. The depth range can be adjusted by changing the number of the pupil zones. In the DH-PSF, the defocus corresponding to half a circle rotation of the double helix is proportional to *Lπ*. The results shown in Fig. 3(d) suggest that the iDH-PSFs possess the similar property. Additionally, the pattern of the iDH-P5 is more stable in comparison to that of the iDH-P3, in particular when it rotates over a larger angle, which corresponds to a larger depth of defocus. This means that the imaging range provided by the iDH-P5 is larger. Indeed, it reaches 3 *μ*m in the configuration that we studied, which is about three-fold of that can be obtained by other interferometric-localization-based super-resolution imaging methods.

**Figure 4:**
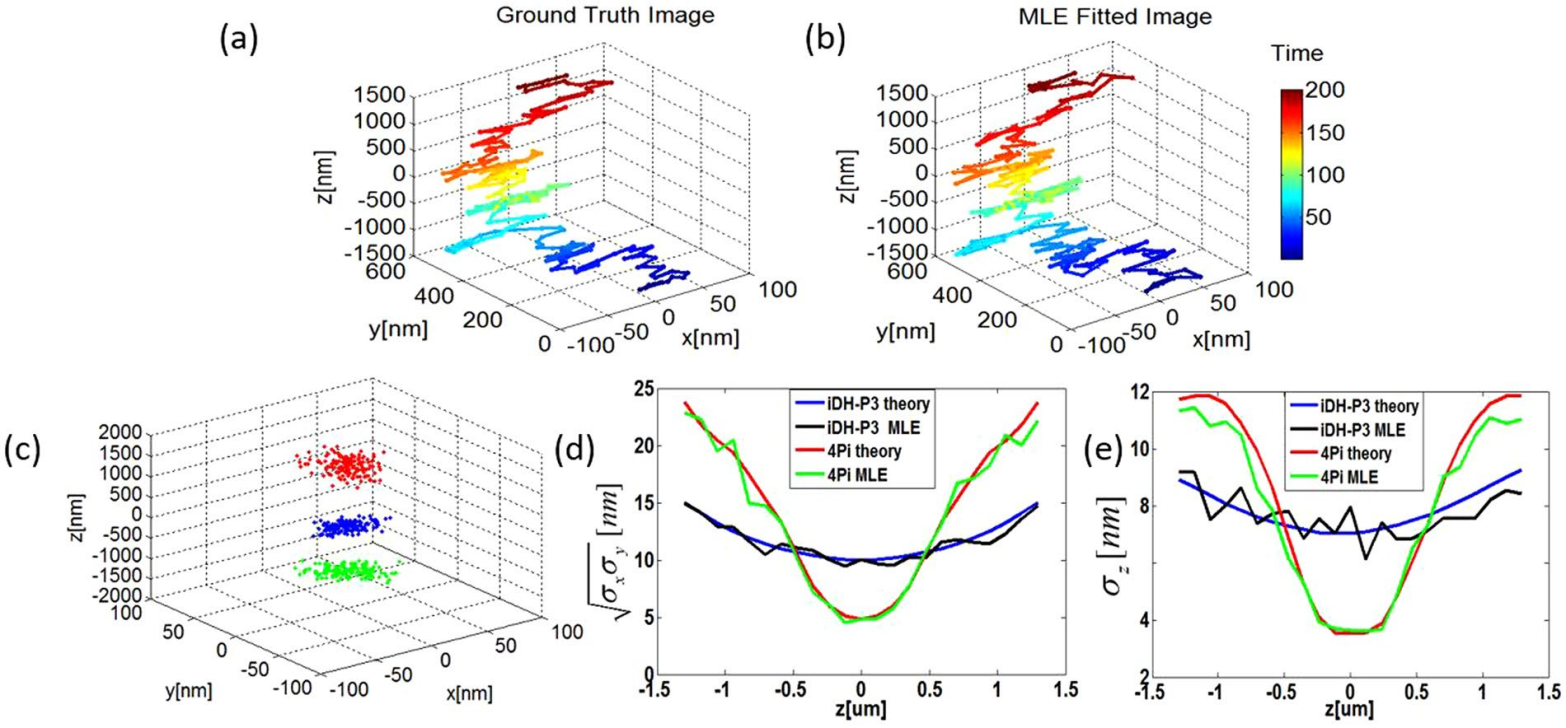
(**a**) Ground-truth trajectory of a single emitter, and (**b**) the reconstructed trajectory using the proposed interferometric-based system with a iDH-P3 PSF; (**c**) The reconstructed point clusters corresponding to three points with the ground-truth position at (0 nm, 0 nm, −940 nm) (red), (0 nm, 0 nm, 0 nm) (blue), and (0 nm, 0 nm, 1300 nm) (green), respectively. (**d**,**e**) Comparisons of the proposed iDH-P3 PSF with the 4Pi in terms of estimation errors in the transverse (**d**) and axial (**e**) direction.

To demonstrate the capability of extending the depth of field of the proposed iDH PSF, we applied it to a realistic simulated data set, which is shown in Fig. 4(a). It represents the trajectory of a single emitter which follows a biased random move towards the +*z* direction. In the trajectory, the emitter moves between a large depth range [−1500 nm, +1500 nm]. In the simulation, we demonstrate the tracking of the emitter by the proposed method, taking the iDH-P 3 PSF as an example. For each position of the emitter in the trajectory, one image was ‘captured’ using the system shown in Fig. 1 with the iDH-P3 PSF. This was calculated by the maximum likelihood estimation (MLE). In the simulation, the size of the image had the dimension of 41 × 41 pixels, each of which was of 5.6 *μ*m × 5.6 *μ*m in size. The down sampled factor is 4, meaning that one pixel was actually composed of 4 × 4 sub-pixels. The simulated coherent PSF was generated using the chirp z transform (CZT)^24^ in order to accelerate the simulation procedure. The photon count for each pixel was calculated from the simulated coherent PSF using the Poisson random number generator. The MLE fitted trajectory reconstructed from all the images captured by the system is shown in Fig. 4(b). Due to the large depth of field that the iDH-P 3 PSF can offer, the fitted trajectory is very similar to the ground truth one.

To quantify the estimation error, we simulated to capture 150 images of the emitter at each axial position. Thus, for every depth value, we reconstructed a cluster of positions containing 150 points. A typical plot of point cluster is shown in Fig. 4(c), which is corresponding to three set of position cluster with the ground-truth position at (0 nm, 0 nm, −940 nm) (red), (0 nm, 0 nm, 0 nm) (blue), and (0 nm, 0 nm, 1300 nm) (green), respectively. The estimation errors in the transverse and the axial directions are shown in Fig. 4(d) and (e) (black lines), respectively. The simulation results suggest that both the transverse $(\sqrt{{\sigma }_{x}{\sigma }_{y}})$ and the axial estimation error (*σ*_*z*_) are well consistent with our theoretical prediction (blue lines). We also plot in Fig. 4(d) and (e) the localization precision of the same set of ground-truth data estimated by using the 4Pi microscope for comparison. Again, the results are consistent with the theoretical prediction shown in Fig. 3.

## Methods

### Calculating the CRLB

The PSF collected on each camera is defined as *I*_*k*_ (*k* = 1, 2, 3, 4). When estimating the CRLB, we use the intensity images *I*_1_, *I*_2_, *I*_3_, *I*_4_ collected by the cameras. The model for calculating the CRLB is the same as the work in ref. 13. For every channel, the photon detection is an independent Poisson procedure. Details of the model are as follows:

Step 1: Normalize the detection PSFs:

$$\sum _{k}\int {\rm{d}}x{\rm{d}}y{I}_{k}(x,y)=1.$$

Step 2: Calculate the Fisher information matrix for each detection channel as:

$${F}_{k}(i,j)=\sum _{p=1}^{{N}_{p}}\frac{{N}^{2}}{N{I}_{k}(p)+b}\frac{\partial {I}_{k}(p)}{\partial i}\frac{\partial {I}_{k}(p)}{\partial j},$$

where *i*, *j* represent the spatial coordinates (*x*, *y*, *z*), *N* the number of photons collected by each objective lenses, *b* the homogeneous background photons per pixel, and *N*_*p*_ the number of the pixels.

Step 3: Sum up the Fisher information for each detection channel and yield the final Fisher information matrix:

$$F(i,j)=\sum _{k}{F}_{k}(i,j).$$

Step 4: The final localization precision is calculated from the square roots of the diagonal elements of the inverse of the Fisher matrix as:

$${\sigma }_{i}=\sqrt{{F}^{-1}}.$$

Note that both the DH-P *L*-PSF and iDH-P *L*-PSF have a spatial symmetry, i.e., *I*_*k*_ (*x*, *y*) = *I*_*k*_ (−*x*, −*y*), we have the following relationships between the derivative of the intensity images: $\frac{d{I}_{k}}{dx}(x,y)=-\frac{d{I}_{k}}{dx}(-x,-y)$, and $\frac{d{I}_{k}}{dz}(x,y)=\frac{d{I}_{k}}{dz}(-x,-y)$. This means that the off-diagonal elements between the transverse and the axial localization is 0. But there are a slight correlation between the transverse localization precision matrix.

### Demonstration of the overlapping between the two PSFs

For an optical system with two objective lenses, the two PSFs collected by the objective lenses have the following form:

6 $$\begin{array}{rcl}{E}_{1}({\rho }_{p},z,{\varphi }_{p}) & = & -\frac{ik}{2\pi }{\int }_{0}^{2\pi }{\rm{d}}\varphi {\int }_{0}^{{\rm{\Theta }}}{\rm{d}}\theta {T}_{1}(\theta ,\varphi )\sin \,\theta \sqrt{\cos \,\theta }\exp (ikz\,\cos \,\theta )\\  &  & \times \,\exp (ik{\rho }_{p}\,\sin \,\theta \,\cos \,({\varphi }_{p}-\varphi ))\end{array}$$

7 $$\begin{array}{rcl}{E}_{2}({\rho }_{p},z,{\varphi }_{p}) & = & -\frac{ik}{2\pi }{\int }_{0}^{2\pi }{\rm{d}}\varphi {\int }_{0}^{{\rm{\Theta }}}{\rm{d}}\theta {T}_{2}(\theta ,\varphi )\sin \,\theta \sqrt{\cos \,\theta }\exp (-ikz\,\cos \,\theta )\\  &  & \times \,\exp (ik{\rho }_{p}\,\sin \,\theta \,\cos \,({\varphi }_{p}-\varphi ))\end{array}$$

For the two mutual-conjugated annular pupil functions, the coherent PSFs can be written as

8 $${E}_{1}({\rho }_{p},z,{\varphi }_{p})=-\sum _{l=1}^{L}{i}^{2l+1}k{\int }_{{\theta }_{l}}^{{\theta }_{l+1}}{\rm{d}}\theta \,\sin \,\theta \sqrt{\cos \,\theta }\exp (i(kz\,\cos \,\theta -2l{\varphi }_{p})){J}_{2l}(k{\rho }_{p}\,\sin \,\theta )$$

9 $${E}_{2}({\rho }_{p},z,{\varphi }_{p})=-\sum _{l=1}^{L}{i}^{2l+1}k{\int }_{{\theta }_{l}}^{{\theta }_{l+1}}{\rm{d}}\theta \,\sin \,\theta \sqrt{\cos \,\theta }\exp (-i(kz\,\cos \,\theta -2l{\varphi }_{p})){J}_{2l}(k{\rho }_{p}\,\sin \,\theta )$$

Note that we have ${E}_{1}=-{E}_{2}^{\ast }$, which promise perfect overlap between the two coherent PSFs. In the interferometric rotation PSFs, perfect overlap ensures that the interferometric effect occurs in the azimuthal direction, which is not achievable by using other kinds of DH-PSFs.
